# Everyday ethics of suicide care: Survey of mental health care providers’ perspectives and support needs

**DOI:** 10.1371/journal.pone.0249048

**Published:** 2021-04-22

**Authors:** Marjorie Montreuil, Monique Séguin, Catherine P. Gros, Eric Racine

**Affiliations:** 1 Ingram School of Nursing, McGill University, Montreal, Quebec, Canada; 2 Quebec Network on Suicide, Mood Disorders and Related Disorders, Douglas Mental Health University Institute, Verdun, Quebec, Canada; 3 Institut de recherches cliniques de Montréal, Montreal, Quebec, Canada; 4 Department of Psychology, Université du Québec en Outaouais, Gatineau, Quebec, Canada; 5 Départements de Médecine et Médecine sociale et préventive, Université de Montréal, Montreal, Quebec, Canada; 6 Departments of Neurology and Neurosurgery, Medicine, and Biomedical Ethics Unit, McGill University, Montreal, Quebec, Canada; Imam Abdulrahman Bin Faisal University, SAUDI ARABIA

## Abstract

Suicide occurs in people of all ages and backgrounds, which negatively affects families, communities, and the health care providers (HCPs) who care for them. The objective of this study was to better understand HCPs’ perspectives of everyday ethical issues related to caring for suicidal patients, and their perceived needs for training and/or support to address these issues. We conducted a mixed methods survey among HCPs working in mental health in Québec, Canada. Survey questions addressed their perspectives and experiences of everyday ethical challenges they encounter in their practice with people who are suicidal, and their perceived needs for training and/or support therein. 477 HCPs completed the survey. Most participants mentioned encountering ethical issues when caring for people who are suicidal. The challenges HCPs encounter in their practice with people who are suicidal are numerous, including issues related to maintaining privacy, confidentiality, freedom and the therapeutic relationship. The lack of time, resources and professional support to address these issues was emphasized. Most HCPs reported that the training or education they have received does not allow them to address everyday ethical issues related to suicide care. In sum, there is a clear reported need for better training and support for HCPs who are offering care to people who are suicidal in relation to everyday ethical issues they encounter. Implications for practice include providing greater access to training, including access to specialists in ethics to address specific issues. This additional support could alleviate morally distressing situations for HCPs.

## Introduction

Suicide accounts for the death of over 800,000 individuals worldwide per year, making it an international public health issue [1]. Though consistently under-reported and often misclassified, deaths by suicide occur in people of all ages and backgrounds, which has a major impact on their families, communities, and the health care providers (HCPs) who care for them [2,3]. In Canada, it is estimated that for each death by suicide, there are 25–30 additional suicide attempts, many of which result in emergency department visits, direct hospitalization, and/or mental health facility admissions [4–6]. The presence of suicidal patients in various healthcare settings has important ethical implications for the HCPs caring for these individuals and their families [7].

HCPs are often the first point of contact for patients following a suicidal episode, and thus play a crucial support role in suicide assessment, prevention and management [8–11]. Knowledge about suicidal behavior has increased greatly in recent decades, with abundant literature on suicide risk assessment and prevention strategies [2]. More recent studies have examined the lived experiences and perceptions of various HCPs caring for suicidal patients [11–16]. These studies highlight the various emotional and ethical challenges that accompany caring for this patient population. Two recurrent themes include HCPs desiring additional training on suicide care and the lack of evidence-based clinical guidelines to assist HCPs in suicidal patient management [7,16].

Multiple studies have further indicated the benefits of additional educational training and guidelines on suicide management for HCPs, including their improved understanding, knowledge, willingness, attitudes, and confidence in caring for suicidal patients [10,15,17,18]. These outcomes have the potential to improve the quality of mental health care service delivery to suicidal patients. However, few guidelines and frameworks currently exist to guide in dealing with ethical aspects of suicide care [7,16].

### Everyday ethics related to suicide care

Everyday ethics refers to the ethically charged situations that arise in day-to-day clinical practice [19]. In suicide prevention care, various ethically charged situations have notably been identified [7]. In a literature review on the ethical challenges of suicide care, three broad categories of ethical issues were identified: (1) ethical issues arising from discrete decisions and acute care settings, (2) ethical issues arising from therapeutic relationships and chronic care, and (3) organizational factors and their effect on care [7]. Across all three categories, potential everyday issues were identified, including involuntary hospitalization, therapeutic relationships between HCPs and suicidal patients, and issues in the training and preparation of HCPs to treat suicidal patients (Table 1) [7]. Acknowledging these issues could facilitate their clinical management and ensure they are properly addressed in health education programs [19,20].

**Table 1:** Ethical issues identified within the literature on suicide and clinical care [7].

Category	Definition	Topics Included in the Questionnaire
Ethical issues arising from discrete decisions and acute care settings	Primarily standalone events that require healthcare professionals to make a binary choice (yes/no or permissible/impermissible)	• Involving surrogate decision-makers • Involuntary hospitalization
Ethical issues arising from therapeutic relationships and chronic care	Factors and contexts that influence the care that healthcare professionals provide to suicidal patients and that emerge during extended periods of treatment	• The value of therapeutic relationships and important factors in their development • Different clinical responses needed when treating a chronically suicidal person versus an acutely suicidal person • The impact of suicidality on the well-being of healthcare professionals
Organizational factors and their effect on care	Organizational or institutional factors that impact healthcare professionals’ ability to provide care	• Training and preparation of healthcare professionals to treat suicidal patients

Traditional principles of bioethics, namely autonomy, beneficence, nonmaleficence, and justice have the potential to become confounding when applied to the everyday ethics of suicide care [21,22]. For example, HCPs’ duty to do no harm (nonmaleficence) can contradict a suicidal patient’s autonomy regarding end-of-life decision-making, leading to ethical dilemmas for HCPs. Addressing the educational and support needs of HCPs could positively impact the care and management of suicidal patients and the quality of mental health services [15].

In order to address this overlooked topic, we conducted a survey among HCPs working in mental health regarding their perspectives and experiences of the everyday ethical issues they encounter in their practice with patients who are suicidal and have a mental health disorder. The aim of this survey was to better understand HCPs’ perspectives of everyday ethical issues related to caring for suicidal patients, and HCPs’ perceived needs for training and/or support to address these issues.

## Materials and methods

A mixed method embedded survey approach was used, for which we developed a questionnaire that included closed and open-ended questions [23]. The questionnaire had six sections, four of which were pertinent to this article: (1) demographic information; (2) practices related to suicide care; (3) everyday ethics in relation to suicide care; and (4) training needs to address ethical issues related to suicide care. Other, deliberately separate sections of the questionnaire addressed medical-aid-in-dying in the context of a mental illness. Two corresponding general and distinct research questions guided these different sections: one of which is addressed here in relation to suicide, and the other in relation to medical-aid-in-dying for which the results are published elsewhere [24]. The methods and analysis sections for these two articles are therefore similar but address different research questions and present two separate datasets. The close-ended questions inquired whether how or if participants frequently had experienced the ethical challenges associated with suicide care described in the literature (Table 1), as well as additional issues identified in consultation with an interdisciplinary working group (composed of four HCPs and one ethicist). Each close-ended question was followed by an open-ended question inviting participants to elaborate on their replies (e.g., rationale for their choice; explanation; examples from their practice). We also collected demographic information about HCPs’ profession or job title, work environment, age, years of practice, previous formal ethics training or education they have received, and religious/spiritual beliefs (see Supporting documentation for a copy of the questionnaire with the sections relevant to this study). By the term HCPs, we refer to individuals who are offering mental health services as part of their work for an institution or organization, for example nurses, psychologists, social workers, physicians and psychoeducators. In Québec, psychoeducators are professionals who provide assessments and interventions for people who present with adaptation or behavioral issues [25].

### Pilot-testing

Six mental health experts offered feedback on the questionnaire about clarity, content, understanding of the questions, and willingness to respond [26]. We then conducted two workshops with members of a mental health team (lasting 75 minutes each) to further refine the questionnaire. In each workshop, we asked questions to the participants regarding the clarity of the questionnaire and consent form, their relevance, the participant’s willingness to answer the questions, suggestions of questions to include, the participant’s preferred format and length, and other suggestions for improvements. The questionnaire was pilot-tested in both French and English.

### Data collection procedures and data analysis

The study received approval from the Research Ethics Board of the Institut de recherches cliniques de Montréal, in accordance with the Canadian Tri-Council Policy Statement principles. Informed consent was obtained from each participant before participating to the study. Seven professional associations within Québec agreed to disseminate the questionnaire to their members, representing the following disciplines: medicine, psychiatry, nursing, psychology, psychoeducation and social work. We were targeting HCP who self-declared as working with people who have a mental health disorder. The questionnaire was available online (SurveyMonkey) or on paper upon request, for a period of four months. Data received in paper form were entered online by a research team member.

Quantitative analyses were conducted with SPSS for windows Version 20 by a statistician and were double-checked for accuracy by a research assistant skilled in quantitative analysis. Frequencies were computed for categorial questions and descriptive statistics (mean and standard-deviations) were assessed for continuous questions. Differences between groups based on profession, age, and level of professional experience were evaluated with chi-square statistics (categorical questions) and univariate analysis of variance (continuous questions). Odds ratio (OR) and their 95% confidence intervals (95% CI) were calculated for significant chi-square statistics. In some groups, the number of respondents were too low and/or the sample size per answer was too small to test for differences (for example, for the analyses by profession, only the categories nurses, psychoeducators, and psychologists could be included). This was sometimes exacerbated by the fact that it was possible to skip questions, which also lowered the sample size per answer.

Qualitative analyses were conducted using a coding process [27]. An Excel matrix incorporating all identified codes was created. We then compared and contrasted the codes to identify themes. Quantitative and qualitative data sets were combined using a concurrent mixed-methods framework [23].

## Results

In total, 477 HCPs working in mental health in the province of Québec, Canada, completed the questionnaire. The respondents included in the general descriptive statistics are those, of the 477, who answered these specific questions. The socio-demographic and professional profiles of the respondents are represented in Table 2. For the inferential statistics regarding profession, age, and level of professional experience, the number of respondents is indicated in Table 2.

**Table 2:** Respondents socio-demographic and professional profiles.

		Frequencies	
		Percentages, %	(n)^a^
**Profession**	Nurses	34.4	(159)
	Psychologists^**a**^	24.3	(116)
	Psycho-educators	24.0	(111)
	Social workers	6.71	(32)
	Social interveners	2.8	(13)
	Specialized educators	1.1	(5)
	Occupational therapists	1.3	(6)
	Physicians	0.6	(3)
	Nursing assistants	0.2	(1)
	Patient care attendants	0.2	(1)
	Other	3.2	(15)
**Workplace**^**b**^	Public services in the community	23.9	(114)
	Private practice	19.1	(91)
	External clinic	17.2	(82)
	General psychiatric unit	14.7	(70)
	Specialized psychiatric unit	13.2	(63)
	Community organization	8.80	(42)
	Other	19.5	(93)
**Age (in years)**	18–24	3.70	(17)
	25–34	22.1	(102)
	35–49	42.6	(197)
	50–64	26.6	(123)
	65+	5.00	(23)
**Years of professional experience**	0–5	19.0	(88)
	6–10	18.4	(85)
	11–15	19.0	(88)
	16–20	12.3	(57)
	20+	31.2	(144)

^a^ The total does not always match the number of respondents, as some respondents did not answer certain questions.

^b^ Respondents could choose more than one workplace for this question.

### Experiences with the care of suicidal people

A large majority of respondents had provided care to people at risk for suicide (94.7%; 376/397). Of those, a little less than one third (28.8%; 109/378) cared for suicidal persons during involuntary hospitalization on at least three occasions per month and 20.4% (77/378) worked with this subgroup on a weekly basis. A significant association was found across professional groups (χ^2^(4) = 35.63, p < .0001), with nurses being much more likely to care for suicidal persons during involuntarily hospitalization compared to psychoeducators (OR = 5.54, 95% CI: 3.07–9.99) or psychologists (OR = 11.72, 95% CI: 6.27–21.9;). No associations for care during involuntarily hospitalization were found based on respondents’ age or levels of professional experience.

While most HCPs had contact with close friends and relatives of suicidal persons (86.6%; 344/397), a significant association was present across professional groups (χ^2^(2) = 21.21, p < .0001). Nurses again were more likely than psycho-educators (OR = 1.89, 95% CI: 0.72–4.98) and psychologists (OR = 5.71, 95% CI: 2.46–13.26) to encounter friends and relatives of suicidal people, and psychoeducators more so than psychologists (OR = 3.02, 95% CI: 1.37–6.68).

### Ethical challenges encountered with the care of suicidal people

#### Ethical issues arising from discrete decisions and acute care settings

The main ethical challenges involved two distinct areas of care: involuntarily hospital admissions and the involvement of close friends and relatives. For approximately one third (35.7%; 135/378) of respondents, caring for suicidal patients during involuntarily hospitalization was ethically challenging (e.g. related to the ‘involuntary’ aspect of care or difficulty in maintaining a therapeutic relationship). Roughly one quarter (27.7%; 110/397) of respondents reported that encounters with friends and relatives of suicidal clientele were a source of ethical difficulty—a challenge they experienced up to three times a month (e.g. related to what information can be shared, or the friends and relatives’ distress). Of the HCPs who encountered regular difficulties (> than once a week) related to the involvement of close friends and relatives (5.3% of respondents; 21/397), the majority (over 85%) did not provide care to suicidal patients on a daily basis. In contrast, the HCPs who reported monthly difficulties provided care to people at risk of suicide on a daily basis 65% of the time.

Significant differences according to professional groups were detected for the two ethical challenges previously mentioned: for the involvement of close friends and relatives (χ^2^(4) = 32.86, p < .0001) and in regard to offering care to suicidal people who are involuntarily admitted to the hospital (χ^2^(4) = 27.07, p < .0001). Specifically, nurses were more likely than psychoeducators (OR = 2.48, 95% CI: 1.42–4.33) and psychologists (OR = 5.5, 95% CI: 2.9–10.46) to report regular or occasional difficulty regarding the involvement of family members and friends, and were also more likely than psychoeducators (OR = 2.00, 95% CI: 1.1–3.61) and psychologists (OR = 5.07, 95% CI: 2.66–9.65) to report that they found caring for suicidal people during involuntarily hospitalization ethically challenging. Of note, data also show that nurses were much more likely to be involved in these situations than other work categories.

#### Ethical issues arising from therapeutic relationships and chronic care

Regarding the development of therapeutic relationships with suicidal persons, 18.9% (75/397) of respondents sometimes (up to three times per month) found this difficult while an additional 6.3% (25/397) had difficulty carrying out this fundamental aspect of care on a regular basis (at least weekly) (e.g. clients not sharing information for fear of involuntary hospitalisation). No significant differences across professions were present (χ^2^(4) = 8.15, p >.05). However, significant differences were detected between years of professional experience and ethical challenges related to relationship building (χ^2^(4) = 10.63 p < .05). Respondents with 10 years of experience or less were more likely (OR = 2.06, 95% CI: 1.29–3.31) to report never or rarely encountering difficulties pertaining to the development of a strong therapeutic relationship with suicidal patients, compared to respondents with more than 10 years of experience. However, this might be confounded by other factors such as HCPs changing fields of practice or leaving the profession.

#### Organizational factors and their effect on care

Whether clinicians’ training or education in suicide care adequately prepared them to address the types of ethical challenges they encountered in practice yielded mixed responses, with 41% (151/368) reporting they were sufficiently prepared and 43.2% (159/368) indicating that they were not, while 15.8% (58/368) were uncertain. A similar pattern emerged regarding whether their workplace or institution of employment provided support resources, such as an ethics consultation service (yes: 39.8% (146/367); no: 42.5% (156/367); uncertain: 17.7% (65/367)).

Regarding training, education, or other preparation related to developing therapeutic relationships with people who are suicidal, it was deemed adequate by 61.1% (225/368) of respondents. Regarding the involvement of friends and relatives, 44% (162/368) reported adequate training. For involuntary hospitalization, 39.7% (146/368) indicated that the training received adequately prepared them. Although no differences were detected based on respondents’ age or years of professional experience, one significant difference was found based on respondents’ profession (χ^2^(4) = 35.23, p < .0001). Specifically, nurses were more likely than psychoeducators (OR = 3.5, 95% CI: 1.91–6.42) and psychologists (OR = 3.73, 95% CI: 1.95–7.13) to report that the training or education they received adequately prepared them to address the ethical issues encountered when working with suicidal people during involuntarily hospital treatment.

Considering this, we examined if different types of organizational settings was associated with different types of ethical challenges. We examined if perceived preparation (e.g. training and education) or workplace/institution support (across all respondents) was associated with ethical challenges when caring for a suicidal person related to the development of a strong therapeutic relationship, to confidentiality and privacy protection, the involvement of close friends and relatives, or to offering care to people who are involuntarily admitted to the hospital and are suicidal. We found no significant association between these various ethical challenges and perceived preparation or support (p-values of χ^2^ were all above 0.05).

Respondents’ opinions regarding training and support options are presented in Table 3. Approximately half of respondents reported that access to ethics experts (52.4%), in-person training (47.4%), and team discussions in the workplace (46.8%) were the most appropriate ways to receive training or support to address these ethical issues. In the open-ended questions, the need to have a clear procedure was highlighted, for example in relation to the legal aspects and assessment criteria related to caring for a person who is suicidal.

**Table 3:** Respondents’ perspectives regarding the most appropriate ways to receive training or support about these ethical issues.

	Percentage (%)	N^a^
**In-person delivery**		
Access to ethics experts	52.4	250
In-person training	47.4	226
Team discussions in the workplace	46.8	223
Presentation by an expert (in person)	43.6	208
Group discussion/reflection	42.8	204
Peer support/exchanges	40.5	193
Training capsules (short training sessions in your workplace)	36.5	174
Mentoring	32.3	154
Workshops with vignettes (clinical cases)	31.9	152
Emotional support	29.8	142
**Online delivery**		
Online training	32.3	154
Presentation by an expert (by videoconference)	28.9	138
Online community of practice	14.5	69
**Visual aid**		
Written information (e.g. flyer, articles)	20.3	97

^a^ Respondents could choose more than one way to receive training or support (the sum is therefore larger than 477).

#### Additional ethical challenges

Ensuring the confidentiality and privacy of suicidal persons was the most prevalent reported ethical challenge, with 8.3% (33/397) of respondents indicating that they encountered this difficulty at least once a week and 31.2% (124/397) encountering this difficulty up to three times per month (e.g. related to what information can be shared with friends and relatives or when to break confidentiality in an imminent risk situation). Significant differences across professional groups were detected for this ethical challenge (χ^2^(4) = 34.88, p < .0001). Specifically, nurses more likely than psychoeducators (OR = 2.73, 95% CI: 1.58–4.7) and psychologists (OR = 6.35, 95% CI: 3.41–11.81) to experience regular or occasional difficulty maintaining the confidentiality and privacy of suicidal people.

No association was found between respondents’ age and the number of ethical challenges reported. However significant differences were detected between years of professional experience and ethical challenges related to patient confidentiality (χ^2^(6) = 15.023, p < .05). Respondents with 6 to 10 years of experience were more likely to encounter this challenge at least sometimes (OR = 2.09, 95% CI: 1.23–3.52) compared to those with both more and less work experience.

Other ethical issues included the lack of cohesion within the healthcare team was cited, and the emotional toll of suicide care on clinicians. Regarding interventions for suicide prevention, 41 respondents mentioned the lack of time, resources and professional support as a major concern and 29 experienced difficulties with suicide risk management. The negative impact on relatives when a family member is suicidal was also highlighted as an important issue.

Additional ethical challenges arising for HCPs were identified in response to the open-ended survey questions (which were only briefly mentioned): working with suicidal patients who have comorbidities, who are chronically suicidal, who are children, who feel hopelessness or who lack social support.

## Discussion

Our study found ethical issues related to the care of suicidal patients to be frequent. However, HCPs also reported that the training or education they have received does not allow them to address everyday ethical issues related to suicide care. Psychologists and psychoeducators reported being less well-prepared than nurses to address everyday ethics in relation to suicide care, while almost all participants mentioned encountering ethical issues when caring for people who are suicidal. There is clearly a reported need to offer better training and support for HCPs who are offering care to people who are suicidal in relation to everyday ethical issues they encounter. Otherwise, HCPs may seek to avoid raising the topic of suicide not to have to manage the ethical issues raised [28].

The survey results suggest that nurses are encountering more ethical issues than other HCPs when caring for people who are suicidal. Notably, considering that nurses are likely to be in close contact with relatives and potentially developing therapeutic relationships with them, this relational proximity can lead to confidentiality and privacy issues. A salient example relates to the disclosure of suicidal ideas to family members (or not) and the extent of family members’ involvement in the suicidal person’s care [29]. Also, nurses are in close contact with patients when there is a hospitalization period, being present around the clock on inpatient units, which leads to increased exposure to ethical challenges related to involuntary hospitalization and treatment. Involuntary hospitalization is typically used when a person is considered at imminent risk of suicide, but it directly infringes the person’s freedom, which raises complex ethical issues about the appropriate length and frequency of involuntary hospitalization [30]. Congruent with this heightened presence of reported ethical issues, nurses also mentioned having received the most adequate ethics training in relation to the care of people who are suicidal, but only in regard to involuntary hospitalization. Considering that involuntary hospitalization is a practice regulated by the law, it could explain the increased training provided on this issue by healthcare institutions. Differences in curriculum content during HCP’ education could also explain some of the differences highlighted in the level of comfort by discipline to address ethical issues related to suicide.

The number of years of experience also appeared to affect the reported difficulty in developing strong therapeutic relationships with people who are suicidal. As HCPs gained more clinical experience, it was less challenging for them to develop strong relationships. As described in a recent systematic review, the importance of the therapeutic relationship or alliance between the suicidal person and clinician is key to prevent suicide, especially the suicidal person’s perception of a collaborative relationship [31]. HCP who have more experience may be more skilled at co-developing treatment goals and achieving them, which contributes to the perception of a strong therapeutic alliance by the person [32]. Moreover, it is possible that HCP’s own understanding of the impact of experience on strong therapeutic relationships contributes to the stress experienced by less experienced HCPs who feel less skillful. Considering the central importance of the therapeutic relationship, offering support to novice HCPs (e.g., mentoring) on how to facilitate relationship-building with suicidal people would be warranted. It is also important to realize the value of learning and that the skills acquired with experience have value, which merits to be better recognized by healthcare systems.

### Ethical sensitivity

There could be a greater awareness or management ability of ethical issues from the part of HCPs who have received ethics training or who have more experience, which might reflect a greater ethical sensitivity and capacity to address these issues (e.g. issues related to confidentiality, privacy, restrictions of freedom). Ethical sensitivity has been defined as an “awareness of the ethical implications” of one’s actions within everyday clinical practice [33]. For a HCP, the absence of ethical sensitivity can lead to inaction and incongruent care. It can also lead to greater moral distress, perhaps because of a lack of insight into the nature of moral problems or the lack of ability to deal with them [34,35]. Conversely, the presence of ethical sensitivity can lead to moral agency resulting in ethical action, if the particular context allows for this ethical action to take place. Otherwise, it can lead to moral distress for the HCP [33,36]. By being more aware of the ethical issues that arise, HCPs will likely report experiencing more ethical issues, but also are more concerned as to how to address them and may have more experience in addressing them. Teasing out the relationships between ethical sensitivity, ethics training, and ethics problem resolution skills merits greater attention.

### Implications for ethics training and support for mental healthcare providers

Access to specialists in ethics was presented as the most appropriate way to receive training related to everyday ethics in suicide care, whereas none of the respondents have received this type of training. There is thus a discrepancy between the type of training HCPs perceived as having the potential to be beneficial and what they are currently receiving within the settings where participants work. Access to ethicists is typically perceived more as a punctual consultation service than as a source of training [37] although there are exceptions in how ethics consultations are envisioned [38]. Ethics consultations with experts, especially if they are envisioned and organized to be a learning experience, could be relevant for HCPs to address everyday ethical issues encountered in suicide care, build their skills and appropriate the knowledge gained from these consultations to guide their subsequent practice. Future research on the benefits/limitations of the different types of training HCPs consider helpful would contribute to better tailor the support offered to their reported needs. Additional research on patients’ perspectives of ethical issues encountered would also be relevant. The context of suicide care could be ideal to pursue such research given the acknowledged need for training and the paucity of models available to support suicide care.

### Limitations

For statistical analyses, only the data from HCP’s groups that presented statistically sufficient numbers of respondents were included for comparative analyses. Physicians were noticeably almost absent (3 respondents) despite the dissemination of the questionnaire through physician professional associations, as was done for other groups of HCP, and this represents a limitation of our research. The lack of time could be an issue that prevents Quebec and Canadian physicians to participate in research and a lack of commitment to evidence-based practices [39,40]. Another limitation is that HCPs who completed the questionnaire self-declared working in mental health setting, which could include a variety of interpretations. We however asked participants to identify the specific setting where they worked, which provided more specific information.

Initially, within the questionnaire used for this survey, we also chose to study medical-aid-in-dying in the context of severe and persistent mental illness concurrently with suicide, which could have affected the results and reduced participation given the controversial nature of this issue. This decision was made in alignment with current practices related to medical-aid-in-dying for a mental illness in other countries. This distinction was clearly mentioned in the questionnaire but might have affected the training needs identified.

## Conclusions

This mixed methods survey, conducted among 477 HCPs working in mental health in Québec, is one of the few studies informing on the perspectives and experiences of the everyday ethical challenges in caring for suicidal patients. Issues related to privacy, confidentiality, freedom, the therapeutic relationship, as well as the lack of time, resources and professional support to address these issues are frequent. However, there is a great reported need for ethics training and/or support to address these issues. This additional support could contribute to prevent morally distressing situations for HCPs and improve their own well-being, for them to then better support patients who are suicidal. Within interdisciplinary teams, a better understanding of the shared burden in regard to ethical challenges, and how each co-worker may face them differently based on their professional role as well as role in decision-making, may have an important impact on shared emotional support.

Future research directions could include the study of HCPs’ experiences in more depth, for example through qualitative research methods, to better understand how they experience the ethical issues described and co-construct ways to address them. Studying the perspectives of patients who are suicidal would also contribute to better understand how they experience the situations that give rise to these ethical challenges. It would then be possible to engage in the development of more specific suggestions for HCPs’ training/support that would be anchored in their shared experiences. There is also a great need to develop and test different forms of ethics training to see whether they can support practicing clinicians in useful ways.

## Supporting information

S1 File Eng questionnaire. (PDF)
