# Supplementary material for: Everyday ethics of suicide care: Survey of mental health care providers’ perspectives and support needs
Source: PLoS One. 2021 Apr 22;16(4):e0249048. doi: 10.1371/journal.pone.0249048 (PMC8061990; doi:10.1371/journal.pone.0249048)
Supplement: S1 File — (PDF) [file pone.0249048.s001.pdf]

Questionnaire

Survey eligibility criteria

I am a professional or non-professional working in Quebec with people who have a mental health disorder:

- o Yes
- o No

**I. Demographic information of participants**
1. Profession/job title:<br>o Education psychologist/psychoeducator<br>o Nurse<br>o Nursing assistant<br>o Occupational therapist<br>o Patient care attendant<br>o Physician: specialty________________________<br>o Psychologist<br>o “Intervenant social”<br>o Social worker<br>o Special education teacher<br>o Other: ________________________<br><br>2. Workplace (please indicate the type of setting)<br>N.B.: You can choose more than one reply<br>o Specialized psychiatric unit: _______________________<br>o General psychiatric unit: ________________________<br>o External clinic: _________________________________<br>o Private clinic: ________________________________<br>o Public services offered in the community: _______________________________<br>o Community organization: _________________________<br>o Other: ________________________________<br>3. Age<br>o 18-24 years old<br>o 25-34 years old<br>o 35-49 years old<br>o 50-64 years old<br>o 65 +<br><br>4. Years of work experience with people who have a mental health disorder<br>o 0-5 years<br>o 6-10 years<br>o 11-15 years<br>o 16-20 years<br>o > 20 years

5. Previous training in ethics (Please tick all that apply)
    - Workshops
    - Online training
    - Group discussion/ reflection
    - Written information
    - Presentation by an expert
    - University course
    - Peer support
    - Training specific to medical aid in dying
    - Other: ___________________________
    - None

6. Religious beliefs/ affiliations
    - Religious and practicing: _____________________________
    - Religious and non-practicing: ___________________________
    - Spiritual but not religious: ____________________________
    - Not spiritual and not religious
    - Other
    - Prefer not to respond

7. Do you consider your religious beliefs/affiliations influence your work?
    - Yes
    - No
    - Uncertain
    - Does not apply

II. Reflections regarding ethical issues in everyday practice

In this next section, we address certain dilemmas that can occur during clinical care for people who are suicidal and have mental health problems.

1. **Have you** provided care to people who were suicidal?
   a. Yes
   b. No (proceed to Section 4 on page XX)
   c. Unsure
   d. Prefer not to answer
   Examples
   (optional) :______________________________
   ____

A. Therapeutic relationship with suicidal people

1. **In your practice**, how often do you find it difficult to develop a strong therapeutic relationship with people who are suicidal?
   - o Never
   - o Rarely
   - o Sometimes (maximum three times per month)
   - o Regularly (once a week or more)
   - o Not applicable

   Explanation/examples
   (optional) :______________________________
   ____

B. The involvement of close friends and relatives of a person who is suicidal

2. In your practice, have you been in contact with close friends and relatives of a person who is suicidal?
   - o Yes
   - o No (proceed to Section C)

3. **In your practice**, how often do you encounter difficulties related to confidentiality and privacy protection when caring for a suicidal person?
   - o Never
   - o Rarely
   - o Sometimes (maximum three times per month)
   - o Regularly (once a week or more)

   Explanation/examples
   (optional) :______________________________
   ____

4. **In your practice**, how often do you encounter difficulties related to the involvement of close friends and relatives when caring for a suicidal person?
   - Never
   - Rarely
   - Sometimes (maximum three times per month)
   - Regularly (once a week or more)

   Explanation/examples

   (optional) :____

   ____

C. Care provided to suicidal people who are involuntarily admitted to the hospital

5. How often **have you** been involved with the care of a suicidal person who is involuntarily admitted to the hospital and is suicidal? (Before, during, or after their stay in the hospital)
   - Never (proceed to Section D)
   - Rarely
   - Sometimes (maximum three times per month)
   - Regularly (once a week or more)

   Explanation/examples

   (optional) :____

   ____

6. **Do you consider** offering care to people who are involuntarily admitted to the hospital and are suicidal is ethically challenging?
   - Yes
   - Non
   - Unsure

   Explanation/examples

   (optional) :____

   ____

III. Reflections on training and support needs

1. **Do you consider** that your training or professional education was sufficient in preparing you to address the ethical challenges you face on a daily basis related to people who are suicidal?
   - o Yes
   - o No
   - o Unsure

   Explanation/examples

   (optional) :____

   ____

2. **More specifically, do you consider** that your training, education, or other form of preparation was sufficient in preparing you to address the ethical issues related to:

   a) The development of a therapeutic relationship with people who contemplate suicide?
   - o Yes
   - o No
   - o Unsure
   - o Other (please specify)

   Explanation/examples

   (optional) :____

   ____

   b) The involvement of close friends and relatives of a person with a mental illness who is thinking of suicide?
   - o Yes
   - o No
   - o Unsure
   - o Other (please specify)

   Explanation/examples

   (optional) :____

   ____

   c) The care of people who are involuntarily admitted to the hospital and thinking of suicide?
   - o Yes
   - o No
   - o Unsure
   - o Other (please specify)

   Explanation/examples

   (optional) :____

   ____

3. **Do you consider** that your workplace or institution sufficiently provided you with other types of resources (e.g., an ethics consultation service) to address the ethical challenges you face on a daily basis with people who are thinking about suicide?
   - o Yes
   - o No
   - o Unsure

   Explanation/examples

   (optional) :____

   ____

4. Which of the following are the most appropriate ways to receive training or support about these ethical issues?
   - o Access to ethics experts
   - o Emotional support
   - o Group discussion/ reflection
   - o In-person training
   - o Online community of practice
   - o Online training
   - o Peer support/ exchanges
   - o Presentation by an expert (in person)
   - o Presentation by an expert (by videoconference)
   - o Team discussions in the workplace
   - o Training capsules (short training sessions in your workplace)
   - o Workshops with vignettes (clinical cases)
   - o Written information (e.g. flyer, articles)
   - o Other (please specify):

   ____

   ____

5. What kinds of information would you like to receive about these ethical issues?

   ____

   ____

   ____

   ____

   ____

V. General questions (optional)
1. What other difficult situations have you encountered in your own practice with people who express are thinking about suicide?<br>____<br>____<br>____<br>____<br><br>b) What other ideas/ needs/ comments/ information would you like to share with us?<br>____<br>____<br>____<br>____<br>____
